# Hospital-wide surveillance of central line-associated bloodstream infections in a tertiary hospital in South Korea

**DOI:** 10.1017/ash.2025.120

**Published:** 2025-09-03

**Authors:** Da-Mi Kim, Ji-Young Lee, Dong-Gun Lee

**Affiliations:** 1 Infection Control Office, Seoul St. Mary’s hospitalThe Catholic University of Korea, Seoul, Korea; 2 Division of Infectious Diseases, Department of Internal Medicine, College of MedicineThe Catholic University of Korea, Seoul, Korea

**Keywords:** CLABSI, Hospital-wide Surveillance, MBI-LCBI

## Abstract

**Objectives:** Central Line-Associated Bloodstream Infections (CLABSIs) are associated with prolonged hospitalization, increased healthcare costs. It is important to reduce CLABSI rates through interventions. This study investigated the current status of CLABSI rates among hospitalized patients to gather foundational data for implementing CLABSI intervention measures. **Methods:** During a month from June 1st to 30th, 2023, a retrospective investigation of CLABSI rates was conducted among patients hospitalized at a tertiary hospital in South Korea. Psychiatric and obstetric, hospice, emergency, neonatal wards, and delivery rooms were excluded from the study. CLABSI was defined according to NHSN and Korean National Healthcare-associated Infections Surveillance System. **Results:** A total of 48 CLABSIs were identified, with mucosal barrier injury laboratory-comfirmed bloodstream infection (MBI-LCBI) accounting for 29 (60.4%) and non-MBI-LCBI for 19 (39.6%). Among MBI-LCBI, 28 (96.6%) occurred in hematology wards, while among non-MBI-LCBI cases, 9 (47.4%) occurred in general wards, 9 (47.4%) in hematology wards, and 1 (5.3%) in the intensive care units (ICUs). Overall CLABSI rates was 2.75 per 1,000 catheter days, with 1.66 for MBI-LCBI and 1.09 for non-MBI-LCBI. By department, the CLABSI rates per 1,000 catheter days were 6.11 in hematology wards, 1.02 in general wards, and 0.63 in the ICUs. A total of 58 organisms were isolated, with gram- negative bacteria (78.8%) predominating in MBI-LCBI and gram-positive bacteria (56.0%) in non- MBI-LCBI. Among MBI-LCBI, *Klebsiella pneumoniae* (30.3%), *Escherichia coli* (27.3%) were the most frequently isolated organisms, whereas among non-MBI-LCBI, coagulase-negative staphylococci (16.0%) and *E. coli* (16.0%) were the most frequently isolated organisms. **Conclusions:** The CLABSI rates among hospitalized patients at a tertiary hospital in South Korea was higher for MBI-LCBI than non-MBI-LCBI, with the majority occurring in hematology wards. Since the departments and causative organisms are different depending on MBI-LCBI and non-MBI-LCBI, it is necessary to individualize the CLABSI surveillance policy based on this.

